# Effects of Cadmium on Root Morpho-Physiology of Durum Wheat

**DOI:** 10.3389/fpls.2022.936020

**Published:** 2022-06-23

**Authors:** Erika Sabella, Alessio Aprile, Bernadetta Anna Tenuzzo, Elisabetta Carata, Elisa Panzarini, Andrea Luvisi, Luigi De Bellis, Marzia Vergine

**Affiliations:** Department of Biological and Environmental Sciences and Technologies, University of Salento, Lecce, Italy

**Keywords:** cadmium, *Triticum durum*, heavy metal, phytosiderophore, vesicular trafficking

## Abstract

Durum wheat [*Triticum turgidum* L. subsp. *durum* (Desf.) Husn.] can accumulate a high level of Cd in grains with a significant variability depending on cultivars. Understanding how this toxic element is distributed in cereal tissues and grains is essential to improve the nutritional quality of cereal-based products. The main objective of this work was to investigate roots of durum wheat plants (cv. Iride) exposed to different Cd concentrations (0.5 and 5.0 μM) to identify the mechanisms involved in Cd management. Results showed that the root morphology was altered by Cd treatment both at macroscopic (increased number of tips and primary root length) and ultrastructural levels (cell membrane system damaged, cell walls thickened and enriched in suberin). On the other side, Cd was localized in vesicles and in cell walls, and the metal colocalized with the phytosiderophore nicotianamine (NA). Overall, data suggest that Cd is chelated by NA and then compartmentalized, through vesicular trafficking, in the root thickened walls reducing Cd translocation to the aerial organs of the plant.

## Introduction

Cadmium (Cd) is a well-known heavy metal (HM) widespread in agricultural soils; its compounds are among the most hazardous substances indexed in the priority list of the Agency for Toxic Substances and Disease Registry (2021). Cd affects all life forms because of its high toxicity and solubility in soil and water. It was reported that many kinds of cereal, vegetables, and fruits accumulate Cd, and humans take up at least 90% of Cd originating from plant food (Clemens and Ma, 2016). In leaf tissues, 5–10 μg Cd g^−1^ of total dry matter is harmful to most plants (White and Brown, 2010). Roots absorb Cd, but it localizes to all plant tissues and induces several alterations in plant morphological traits and physiochemical processes. In fact Cd toxicity, in most cases, determines a decrease in root elongation, alterations in root architecture and a reduction in root hair formation (He et al., 2017). The shoot is also adversely affected by Cd in a concentration-dependent manner with an abatement in growth. On the other side, Cd-induced physiochemical changes impacting pigment concentrations, protein levels, fatty acid composition, antagonistic and synergistic effects on nutrient uptake, oxidative defense system and finally, plant hormone levels and photosynthesis activities (Qadir et al., 2014). To prevent Cd accumulation in shoot tissues, some plant species have evolved several mechanisms to reduce the entry of Cd in the xylem and the translocation to the aerial parts: immobilization, compartmentalization and more general stress responses. An important mechanism for the detoxification of Cd-stressed plants is the production of phytochelatins and the subsequent sequestration of Cd-chelates in specific cellular compartments, such as vacuoles, or the development of physical barriers to control Cd movement (Parrotta et al., 2015). Plants that retain HMs in roots are defined as “non-accumulator plants” since, on the contrary, hyperaccumulator plants rapidly translocate HMs to the aboveground organs. *Thlaspi caerulescens* is probably the best Cd/Zn accumulator while the related non-accumulator is *Thlaspi arvense*; in *T. arvense* a significant level of phytochelatins and sequestration into the root vacuoles was associated with a minor root to shoot translocation (Lasat et al., 1998; Ebbs et al., 2002). Guo et al. (2021) also compared the Cd non-hyperaccumulating ecotype (NHE) and the Cd hyperaccumulating ecotype (HE) of *Sedum alfredii*. They found that, under Cd treatment, the thickness of the root cell wall in NHE was twofold higher than in HE and that thickness cell walls of NHE were enriched in low-methylated pectin, which trapped Cd in root, leading to a slight Cd migration into the xylem. Moreover, HE ecotype of *S. alfredii* treated with Cd exhibited a significantly higher hydraulic conductance than the NHE ecotype, which is in accordance with the less extensive suberization associated with a reduced expression of suberin-related genes (Tao et al., 2017). This finding was confirmed by Wu et al. (2019) who pointed out that silicon can reduce Cd concentration in wheat roots (*Triticum aestivum* L. cv. JB Asano) lowering endodermal suberization and in turn by promoting Cd translocation to shoots.

Anyway, literature suggest that a universal mechanism of Cd-tolerance does not exist, and different plant species respond differently to Cd exposure. Human interest in these mechanisms mainly arose from the opportunities to reduce human exposure to high Cd levels. Durum wheat (*Triticum turgidum* L. subsp. *durum* (Desf.) Husn.) could accumulate a high level of Cd in grains, but among durum wheat cultivars, great variability was reported (Arduini et al., 2014; Perrier et al., 2016; Vergine et al., 2017). Iride cultivar was chosen for this experiment since it retains high concentrations of Cd in roots. Moreover, it is one of the most cultivated durum wheat in Italy and it has high adaptability to different environments and abiotic stresses (Vergine et al., 2017).

Furthermore, in previous work, we suggested the key role of the nicotianamine and pathway of the Yang Cycle (for the methionine salvage) in the accumulation of Cd in durum wheat cultivars (Aprile et al., 2018). Here, we investigated the mechanisms by which durum wheat (cv. Iride) retains Cd in roots by analyzing plants exposed to two different Cd concentrations at macroscopic, microscopic and ultrastructural levels, and the involvement of the nicotianamine in the sequestration process. Finally, we investigated the molecular responses of wheat roots with a particular focus on genes involved with HM transport, phytosiderophores synthesis, suberin biosynthesis and vesicular trafficking.

A better understanding of the mechanisms involved in Cd management in wheat may support genetic modifications and breeding strategies to develop safer plants when grown in contaminated soils.

## Materials and Methods

### Growth Conditions and Cadmium Treatment

Plants of durum wheat (cv. Iride) were cultivated hydroponically with three levels of Cd (no cadmium, 0.5 and 5.0 μM CdCl_2_). This commercial cultivar was selected since it is one of Italy’s most cultivated because of its rusticity, adaptability to different environments, and low cadmium translocation to aerial tissues (Vergine et al., 2017).

The grain surfaces were sterilized, and seeds sprouted in *Petri* dishes covered with humified vermiculite. After sprouting (about 1 week in the dark and temperature at 8°C), seedlings were transplanted in cylindric pots (*h* = 50 cm, Ø = 10 cm) filled with perlite, soaked with deionized water, and quickly moved to the hydroponic system as described by Sabella et al., 2021. Three seedlings were planted in each pot, and, for each treatment, three tubes were assembled (three biological replicates). The plants were irrigated with a hydroponic solution at regular intervals (4 h) for 5 min to keep the perlite moistened and avoid stagnation. Plants were grown in the Fitotron® Growth Room (Weiss Technik, United Kingdom) under controlled conditions (Vergine et al., 2017).

The nutrient solution was prepared using reverse osmosis (RO) water (<30 μS cm^−1^) and contained 1.1 mM KNO_3_, 3.0 mM [Ca(NO_3_)_2_ 2H_2_O], 0.2 mM NH_4_NO_3_, 1.2 mM K_2_HPO_4_, 0.04 g/L FeEDDHA, 2.0 mM MgSO_4_, 70 μM H_3_BO_3_, 1.2 μM Na_2_MoO_4_, 1.0 μM ZnSO_4_, 1.0 μM CuSO_4_, and 10 μM MnSO_4_; the pH of the nutrient solution was maintained permanently between 5.5 and 6.0 checking it every 2 days. The hydroponic solution was continuously aerated. Treated plants were cultivated by adding CdCl_2_ to the hydroponic solution to obtain a final concentration of 0.5 or 5.0 μM, continuously from the first day after germination until the sampling date. The 0.5 μM concentration is nontoxic for roots, but agronomically relevant as reported by Harris and Taylor (2013). In fact, plants hydroponically cultivated with this concentration showed different Cd accumulation in grains making it possible to study potential mechanisms of HMs compartmentalization or translocation. The 5.0 μM concentration was tested to evaluate differences in the activated mechanisms for higher Cd concentration.

Root tissues (three for each treatment) were sampled after 15 days from sprouting. Roots were removed from perlite substrate and manually washed to remove the perlite beads from the roots. Samples were washed in reverse osmosis water for 30 s. Root samples for mRNA sequencing were frozen in liquid nitrogen and then harvested at −80°C.

### Root Morphometric Analysis

Root morphology parameters (total root length, the number of tips, average root diameter) were determined using the Root System Analyzer software. This tool analyzes two-dimensional images or image sequences of plant roots. Digital images of roots were obtained with a Nikon D3100 camera (Nikon, Tokyo, Japan). The starting point of the automated root tracing was the creation of binary images from the digital photos with the free tool ImageJ 1.46r.

### Scanning Electron Microscopy

Root samples from plants grown without Cd (control), with 0.5 and 5.0 μM Cd were rinsed in reverse osmosis water and examined employing a scanning electron microscope (SEM). Sections handmade using razor blades were obtained from the region at 10–20 mm from the root-shoot junction which is considered mature tissue (Ouyang et al., 2020) and dehydrated by critical point step, mounted on aluminum stubs and sputter coated with three layers of gold before the SEM observation with a Zeiss EVO HD15 (Carl Zeiss, Jena, Germany) at an accelerating voltage of 15 kV in high vacuum. The thickness of cell walls was measured on SEM micrographs (1,000 and 3,000×) using ImageJ 1.46r.

### Determination of Cd and Mineral Elements

For the determination of Cd and plant micronutrient content, finely ground plant part samples (0.1 g) were dried and digested in a solution containing 6 ml of trace-metal-grade concentrated HNO_3_ and 1 ml of 30% (v/v) H_2_O_2_, in a microwave digestion system Milestone MLS 1200 MEGA (FKV, Sorisole, BG, Italy). Following Massadeh and Snook (2002), 10 ml of deionized water was added after cooling, and the solution was filtered through a Whatman filter paper 40 into a 25 ml volumetric flask. The volume obtained was topped up to the mark with deionized water. Cd was determined by graphite furnace atomic absorption spectroscopy (GF-AAS, PinAAcle, PerkinElmer Analyst 600 System, PerkinElmer, USA) according to Aprile et al. (2018).

### Fluorescence Labeling of Cd in Root

Roots were rinsed in reverse osmosis water and cut with sterile razor blades into segments 5 mm long from the region at 10–20 mm from the root-shoot junction which is considered mature tissue. The root-segment surface was sterilized for 1 min in 70% ethanol and then rinsed three times in sterile distilled water. The cuttings were fixed in 4% paraformaldehyde in phosphate-buffered saline (1x PBS, hereafter PBS) overnight at room temperature, followed by washing in PBS buffer for 10 min at room temperature. After fixation, samples were dehydrated by two successive 1 h incubations in each of 70%, 80%, 95%, and 100% ethanol, then embedded in paraffin and cut into 40 μm-thick sections with a microtome Leica RM 2155 (Leica Microsystems, Mannheim, Germany). Sections were transferred to 1:1 (v/v) PBS: 96% ethanol and maintained at −20°C until staining. To dissolve the paraffin, sections were embedded in toluene for 3 min at 43°C. After removing the toluene, the sections were washed twice for 5 min each with PBS buffer and permeabilized by incubation for 20 min at room temperature in PBS containing 0.5% Triton-X100. After rehydration by ethanolic series (96–70–50%, 3 min each), the localization of Cd in wheat roots was investigated using the Cd-specific fluorescent Leadmium Green AM dye (Molecular Probes, Invitrogen, Carlsbad, CA, United States), according to Tao et al. (2017). Leadmium Green AM dye was prepared according to the manufacturer’s instructions; briefly, a stock solution of fluorescent dye was made by adding 50 ml of DMSO to one vial of Leadmium Green AM. The stock dye solution was then diluted 1:10 with 0.85% NaCl prior to being used. Forty micrometer thin sections were stained in the dark for 75 min and washed three times with saline solution warmed to 37°C. All images were taken on a confocal laser-scanning microscope (Carl Zeiss LSM 700 laser-scanning microscope, Jena, Germany) with excitation at 488 nm and emission at 500–550 nm. All the camera features were set to constant values for each image.

### Transmission Electron Microscopy

Root segments of 5 mm long were excised with sterile razor blades from the region at 10–20 mm from the root-shoot junction which is considered mature tissue; the root-segment surface was sterilized for 1 min in 70% ethanol and then rinsed three times in sterile distilled water. The cuttings were incubated in glutaraldehyde fixative (2.5% in sodium cacodylate buffer 0.1 M, pH 7.4, Sigma Aldrich, St Louis, MA) for 6 h at ice temperature. The samples were washed three times with sodium cacodylate buffer 0.1 M, pH 7.4, each time for 10 min, and then post-fixed in osmium tetroxide (1% in sodium cacodylate buffer 0.1 M, pH 7.4) for 2 h at ice temperature, and washed in sodium cacodylate buffer 0.1 M, pH 7.4 again three times, each time for 10 min. The samples were stained with uranyl acetate (0.5% in water) over night at 4°C. The samples were dehydrated in ethanol (30%, 50%, 70%, 80%, 99% and 100%), for 30 min at each concentration. The samples were embedded in Spurr epoxy resin (TAAB Laboratories Equipment Ltd., Aldermaston, Berks, RG7 8NA, England) and placed in an oven at 60°C for 48 h. Finally, the samples were sliced using an ultramicrotome Leica RM 2155 (Leica Microsystems, Mannheim, Germany) and observed under a TEM Hitachi HT7700 at 80 Kv (Hitachi High Technologies America Inc., Dallas, TX). At least 20 sections for sample from three independent experiments were observed to obtain representative images. The images were analyzed with Hitachi EMIP 5.2 Software.

### Nicotianamine Immunoelectron Microscopy

Until the step of paraffin removal with toluene, sections were obtained following the same protocol as for the fluorescence labeling of Cd with the Leadmium Green AM. After the toluene removal, the thin sections were washed twice for 5 min each with PBS buffer and permeabilized by incubation for 20 min at room temperature in PBS containing 0.5% Triton-X100. After rehydration by ethanolic series (96%–70%–50%, 3 min each), hybridization with a primary antibody against NA (gently provided by the Institute of Plant Genetics and Crop Plant Research of Gatersleben, Germany) was performed at room temperature for 60 min. The specificity of this antibody has been described in detail by Pich et al. (1997). The specificity of the immunostaining procedure was proved by competition experiments using an excess of purified NA (Cayman Chemicals, Michigan, United States) preparation to inhibit the recognition of the endogenous NA in the plant tissue by the NA antibodies (Pich et al., 1997). The sections were then treated with a secondary Alexa Fluor 561 goat-anti-rabbit IgG antibody (Molecular Probes) for 45 min. To study colocalization of NA with Cd, a finally staining with the Leadmium Green AM was performed as reported in the previous paragraph. Images were taken on a confocal laser-scanning microscope (Carl Zeiss LSM 700 laser-scanning microscope, Jena, Germany) with excitation at 561 nm and emission at 603 nm to detect the signal NA-associated; while excitation at 488 nm and emission at 500–550 nm was set to detect the signal Cd-associated.

Images acquisition and data analysis for colocalization were performed with the ZEN software. Colocalization analysis was conducted on a pixel by pixel basis. The software will automatically analyze many different measurements from the scatterplot, and these include the overlap coefficient. The value for the overlap coefficient ranges from 0 to 1; an overlap coefficient with a value of 1 represents perfectly colocalized pixels. In particular, to describe the obtained results of the quantitative colocalization, we used the model proposed by Zinchuk et al. (2013); they produced a set of five linguistic variables (“very weak,” “weak,” “moderate,” “strong,” and “very strong”) tied to the values of the popular colocalization coefficients.

### RNA Extraction and qRT-PCR

Root tissues (grown in control conditions and with Cd 0.5 and 5.0 μM) were frozen in liquid nitrogen, and total RNA was isolated from 0.1 g of samples using TRIZOL (Invitrogen, Carlsbad, United States). cDNA synthesis was carried out using TaqMan® Reverse Transcription Reagents (Applied Biosystems, Foster City, United States) according to the manufacturer’s standard protocol. Amplification reactions were performed using the Applied Biosystems® QuantStudio® 3 Real-Time PCR System. Each reaction consisted of 2 ng of cDNA, 12.5 μl of Power SYBR Green RT-PCR Master mix (Applied Biosystems), 5.0 μM forward and reverse primers, ultrapure DNase/RNase-free water (Carlo Erba Reagents S.r.l.) in a total volume of 25 μl. The cycling conditions were: 2 min at 50°C and 10 min at 95°C, followed by 45 cycles of 95°C for 15 s and 60°C for 1 min. Melting curve analysis was performed after PCR to evaluate the presence of non-specific PCR products and primer dimers.

The primers (Supplementary Table S1) were designed with Primer Express Software 3.0 on the mRNA sequences obtained in previous work (Aprile et al., 2018). The genes tested for the reference gene selection were TIF-6 (contig32233), PP2A (contig26674), UNK (contig10748) from Aprile et al. (2018) and NADH (Ta.9617.1.S1_at) and TIM (Ta.12727.1.S1_at) recovered from the previous work of Aprile et al. (2013).

### Suberin Staining

Forty micrometer-thick sections obtained from roots grown in control conditions and with Cd 0.5 and 5.0 μM were treated until the step of rehydration by ethanolic series (96%–70%–50%, 3 min each), as previously described in the paragraph related to the Leadmium Green AM staining. After the rehydration step, root sections were incubated for 30 min at 70°C in 0.01% (w/v) Fluorol Yellow 088 (Santa Cruz Biotechnology, Dallas, Texas, United States) in lactic acid for suberin staining (adapted by Lux et al., 2005). After staining sections were rinsed in water (three baths of 5 min each) and mounted on slides using glycerol 50% prior to microscope examination. Suberin deposition in cross-sections was observed with a confocal laser-scanning microscope (Carl Zeiss LSM 700 laser-scanning microscope, Jena, Germany) with excitation at 488 nm and emission at 490–540 nm. All the camera features were set to constant values for each image. The intensity of the Fluorol Yellow 088 signal was quantified using the software Image J 1.46r.

### Statistical Analysis

All data were statistically analyzed using one-way ANOVA at a significance level of *p* ≤ 0.05. Single-step multiple comparisons of means were performed *via* Tukey’s *post hoc* test, while Student’s *t*-test was used for simple comparisons against control. Data presented are from three treatments (Control, 0.5 and 5.0 μM CdCl_2_) with three replicates as described above.

## Results

### Root Morphology and Architecture

**Figure 1:**
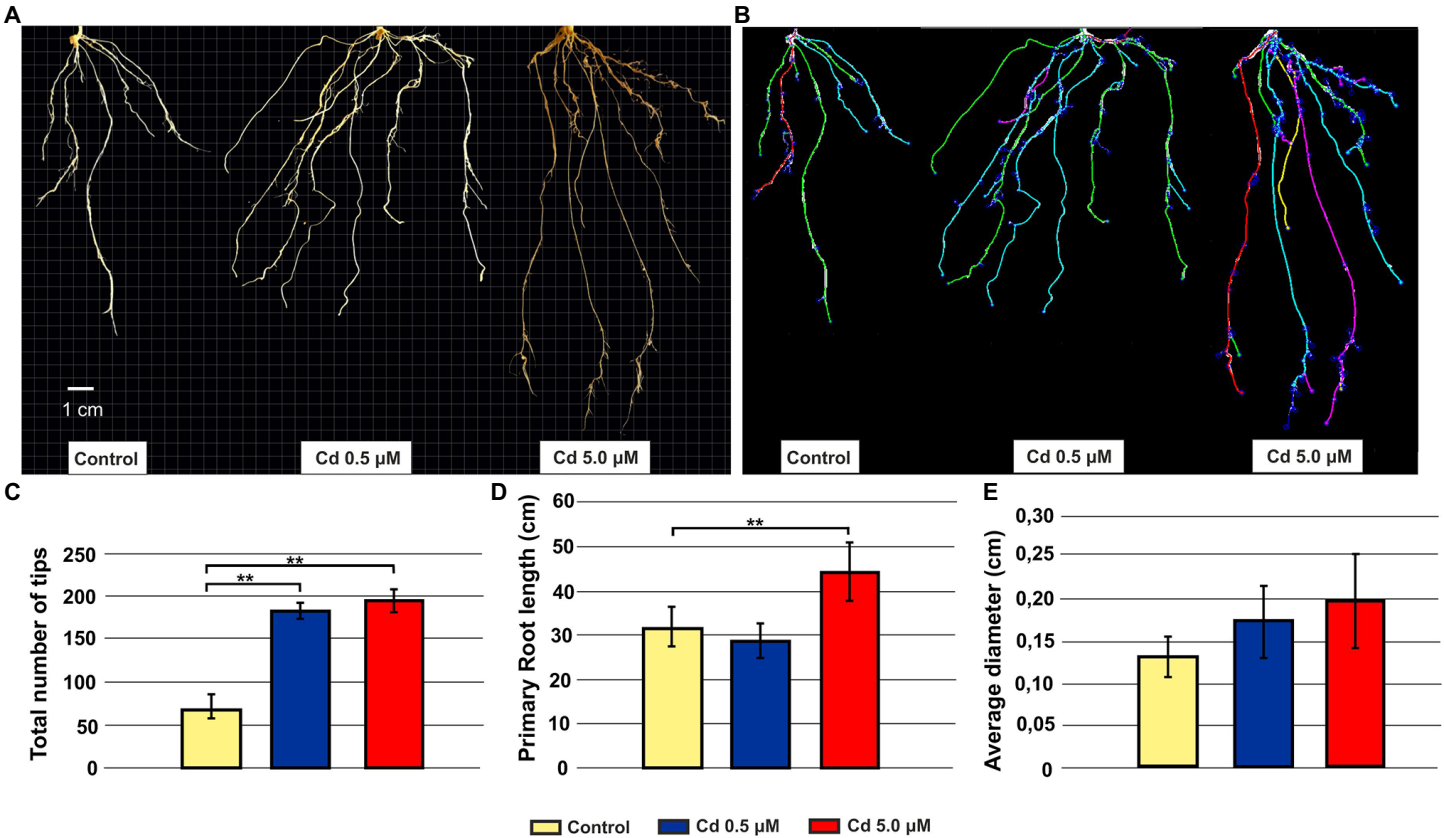
Effect of exogenous Cd on the morphology of roots from seedlings grown in hydroponics for 15 days (starting from sprouting) in the presence of Cd 0.5 and 5.0 μM. Panel **(A)** Representative pictures of roots grown in absence or presence of Cd; panel **(B)** the same root systems of panel A tracked by the image analysis tool Root System Analyzer; panel **(C)** tips total number; panel **(D)** primary root length; and panel **(E)** root average diameter. *T*-test *p* < 0.01**.

Root architecture of plants grown at 0.5 and 5.0 μM Cd revealed a significant increment in the total number of tips compared to the control (Figures 1A–C). A darkening of the roots was observed with increasing Cd concentration to 5.0 μM (Figure 1A); simultaneously, such concentration in the nutrient solution increased the primary root length (Figures 1A,D). No significant variation was detected in the root diameter (Figure 1E).

### Cd Treatments Induced Ultrastructural Changes in Roots

**Figure 2:**
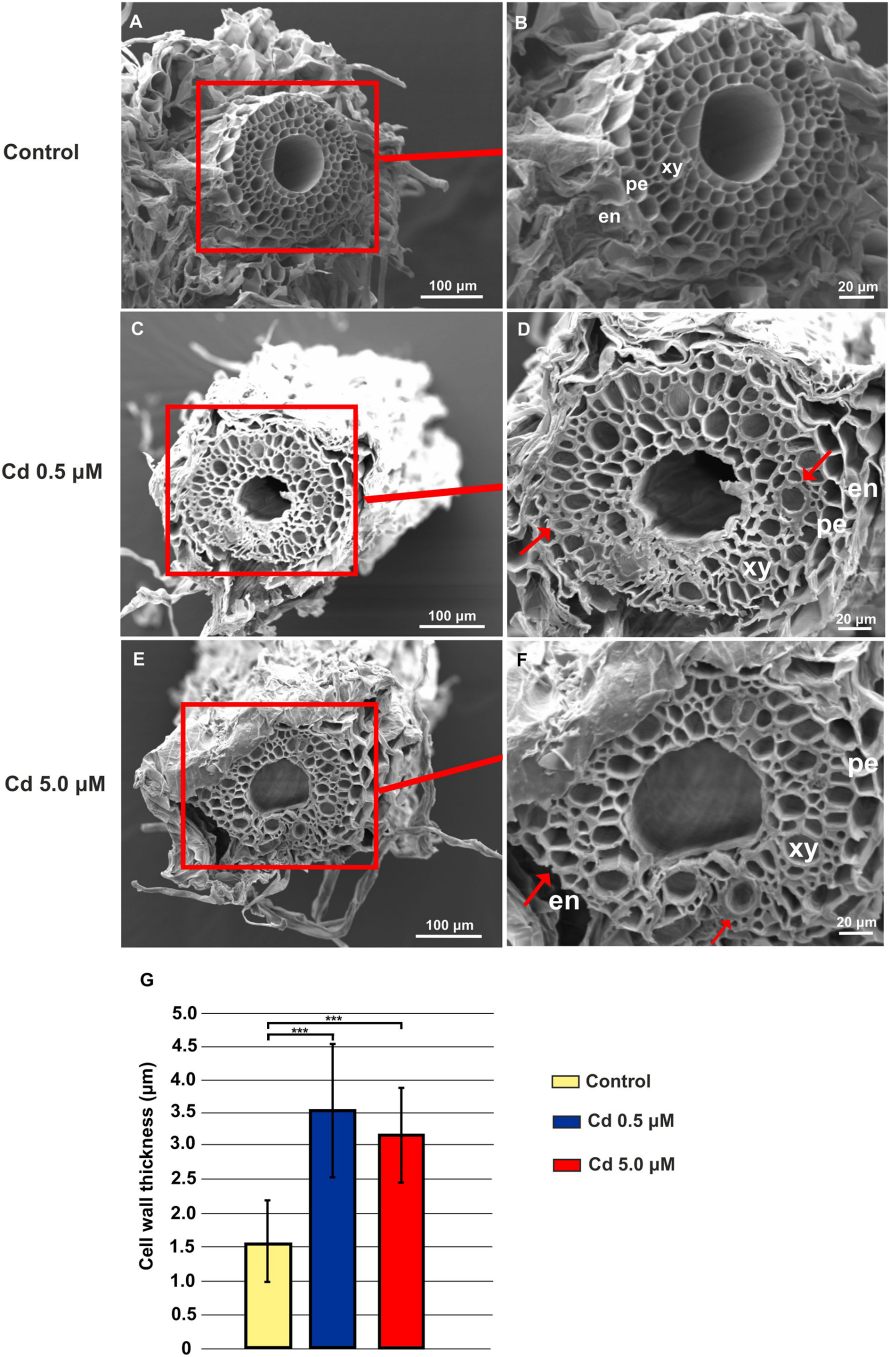
Scanning electron microscope (SEM) microphotographs of root cross-sections obtained from plants grown for 15 days (starting from sprouting) in absence or presence of Cd (0.5 or 5.0 μM). Panels **(A)** and **(B)** Root cross-sections in the control roots; panels **(C)** and **(D)** root cross-sections of plants grown in the presence of Cd 0.5 μM; panels **(E)** and **(F)** root cross-sections of plants grown in the presence of Cd 5.0 μM. en: endodermis; pe: pericycle; xy: xylem. Red arrows indicate pericycle and xylem cell wall thickening. **(G)**, cell wall thickness in the xylem vessels. Eight independent roots were assessed for each treatment (12 cross-sections for root). *T*-test *p* < 0.001***.

Under Cd treatment, considerable ultrastructural anatomical alterations in roots can be observed. Both at 0.5 and 5.0 μM, Cd caused pericycle and xylem cell wall thickening (Figures 2C–F) compared to the structures observed in the cross-sections of roots grown in control conditions (Figures 2A,B). In Figure 2G quantitative data are reported: cell wall thickness, measured in the roots treated with 0.5 and 5.0 μM Cd, was significantly higher (3.54 ± 0.99 and 3.19 ± 0.71 μm, respectively) than in control roots (1.57 ± 0.63 μm; Figure 2G).

### Durum Wheat Plants Store Large Amounts of Cd in Roots

**Figure 3:**
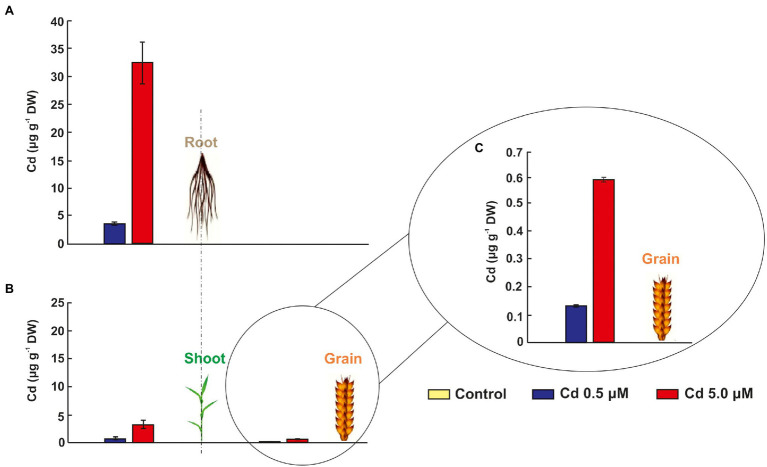
Cadmium in grains, shoots and roots of seedlings grown for 15 days (starting from sprouting) in control conditions and in the presence of Cd 0.5 and 5.0 μM. Panels **(A)** and **(B)** Cd concentrations in μg/g DW (dry weight) of grains, shoots and roots. To appreciate the differences between the two treatments (Cd 0.5 and 5.0 μM) in grains, a third panel was added: panel **(C)** data of Cd concentration in grains.

As expected, the durum wheat plants cv. Iride retains more Cd in the root tissues than the aboveground plant tissues (shoot and grain; Figures 3A–C). In fact, the Cd presence (expressed as μg/g DW) in shoots and grains was five and 26 times and 10 and 53 times lower than in roots, respectively (Figures 3A,B), suggesting a Cd storage in the root. Figures 3A–C show that the Cd level in tissues increases at more elevated Cd concentration, e.g., just under 10 times in case of roots (Figure 3A). Instead, the Cd detected in shoots and grains of plants treated with Cd 5.0 μM was about four times higher than at Cd 0.5 μM (Figures 3B,C).

### Cd Affected the Accumulation of Mineral Elements

**Figure 4:**
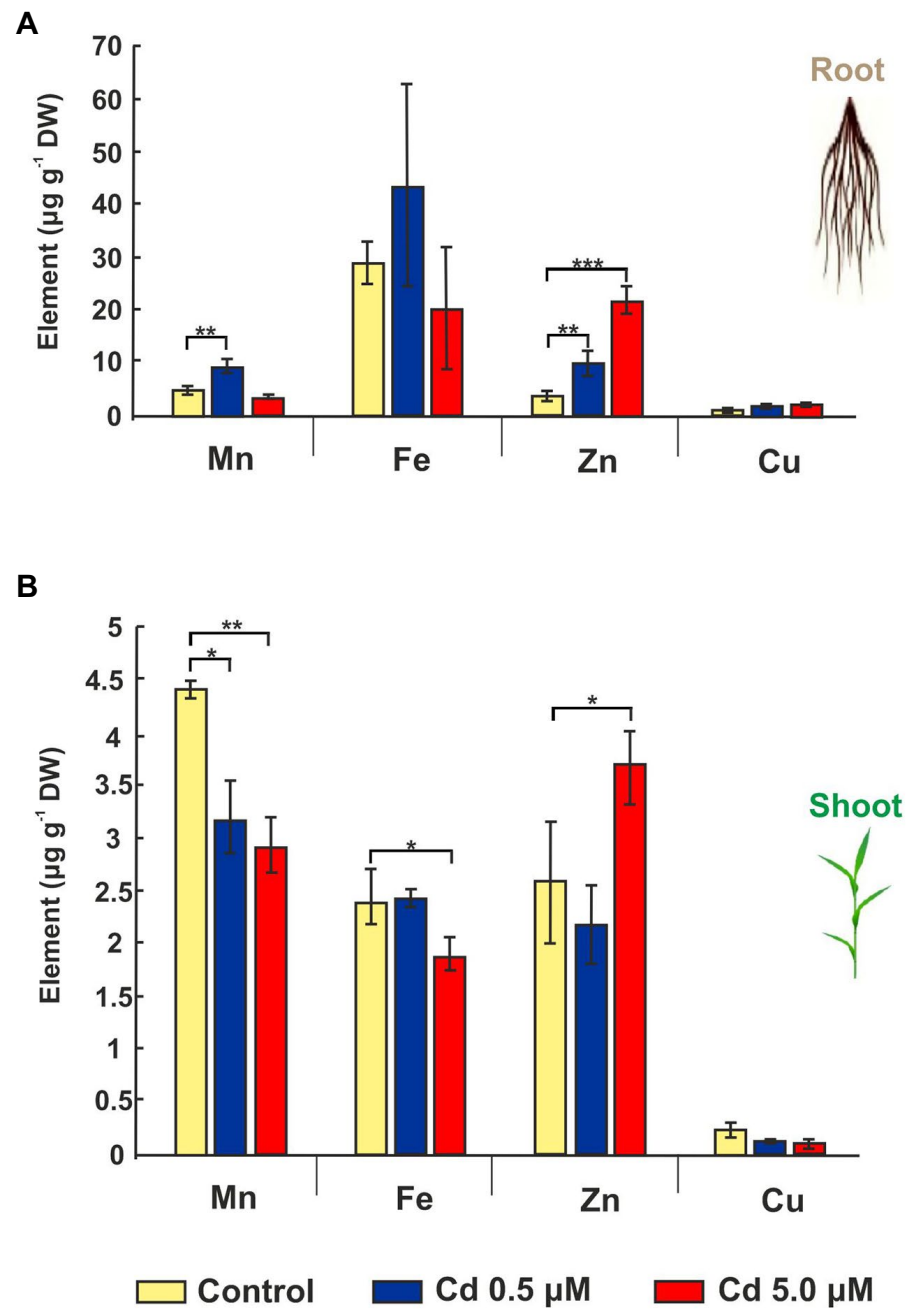
Mineral elements in roots and shoots of wheat seedlings grown for 15 days (starting from sprouting) in control conditions and in presence of Cd 0.5 and 5.0 μM. Panel **(A)** Mn, Fe, Zn, and Cu concentrations expressed as μg/g DW of root. Panel **(B)** Mn, Fe, Zn, and Cu concentrations as μg/g DW of shoot. *T*-test *p* < 0.05*, <0.01**, and <0.001***.

Since Cd treatment can induce iron deficiency that influences root morphology (Jin et al., 2008), the accumulations of other mineral elements were investigated. Cd treatments impacted micronutrient distribution: manganese and zinc significantly increased (+90% and +161%, respectively) for 0.5 μM Cd treatment if compared to control roots (Figure 4A). On the contrary, the 5.0 μM Cd treatment did not affect manganese presence in the root, while the zinc level increased about six times (Figure 4A). The iron amount in the root was not statistically affected by the Cd treatments (Figure 4A). In shoot tissues treated with Cd 5.0 μM, manganese and iron dropped by 33% and 22%, respectively, while an increment (+42%) was detected for zinc (Figure 4B). A treatment with Cd 0.5 μM determined only a decrease of manganese (−27%) if compared to the control, while no significant changes were recorded for iron or zinc content (Figure 4B). Copper did not vary significantly across Cd treatments in root and shoot tissues (Figures 4A,B).

### Distribution of Cd in Wheat Roots

**Figure 5:**
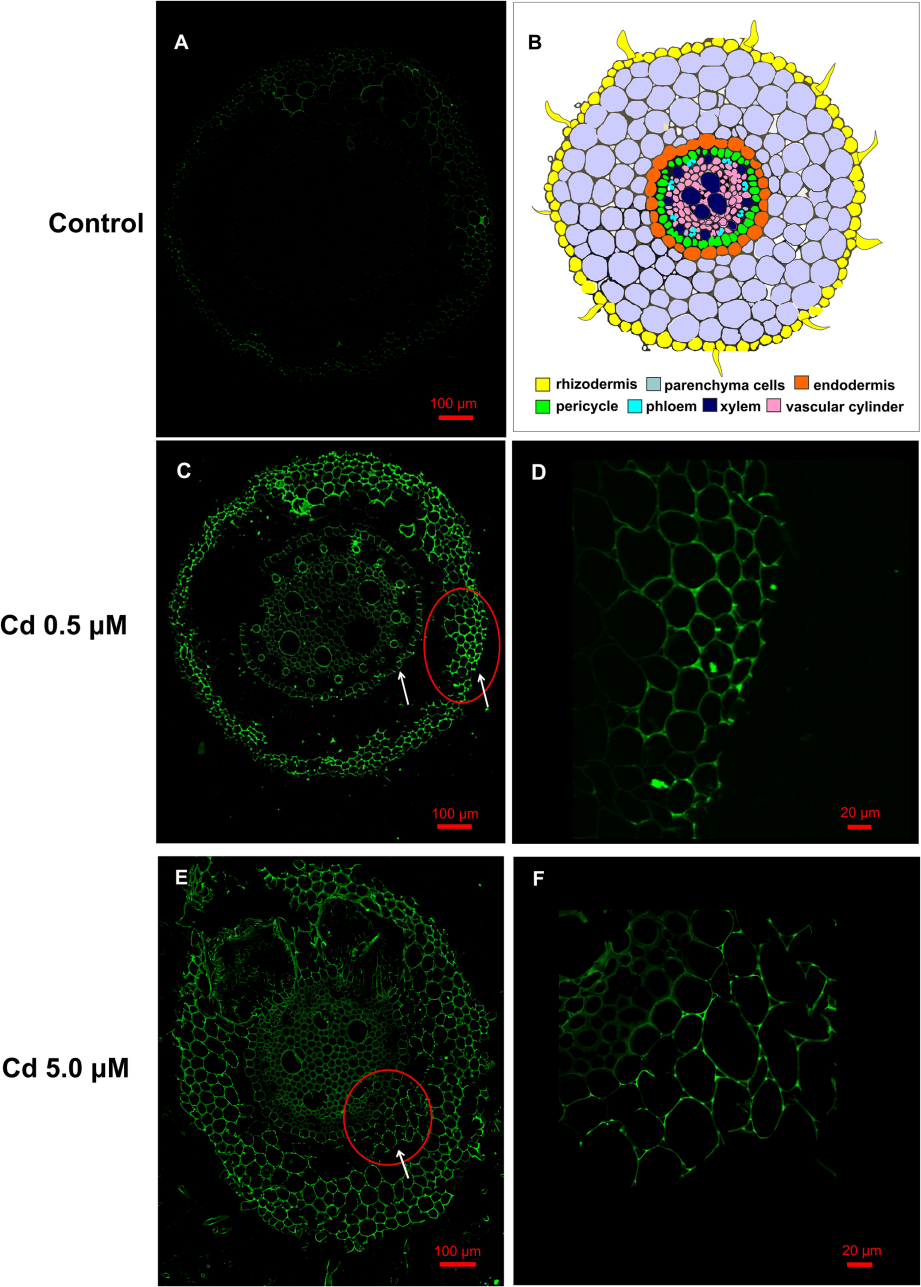
Cd accumulation in roots of the wheat (15 days from sprouting and from the start of Cd treatments) visualized by fluorescent imaging using Leadmium Green AM dye. Green fluorescence represents the binding of the dye to Cd. Panel **(A)** Control roots, a faint but not bright signal is present. Panel **(B)** Schematic overview of wheat root anatomy. Panel **(C)** Roots grown in presence of Cd 0.5 μM, fluorescence is localized in rhizodermis and endodermis (white arrows). Panel **(D)** Detail at higher magnification of the rhizodermis area corresponding to red circle of panel **C**. Panel **(E)** Roots grown in presence of Cd 5.0 μM, fluorescence is also distributed in the cell wall of the parenchyma cells of the cortical region (white arrows). Panel **(F)** Detail at higher magnification of the corresponding to the red circle of panel **E**.

Figures 5A–F show fluorescent microscopic images of the Cd distribution in wheat roots exposed to the two Cd concentrations for 15 days. Except for the image of the control treatment (Figure 5A), in which we can see only a faint but not bright signal (as a result of autofluorescence signal produced by polyphenols and other compounds), in all other images, a bright green fluorescence can be easily observed. In images of roots grown exposed to Cd 0.5 μM, bright green fluorescence was observed in rhizodermis and in endodermis cells (Figures 5C,D). At the 5.0 μM Cd concentration, a bright fluorescence was evident in the parenchyma cell walls of the cortical region (Figures 5E,F). These observations indicated that at low concentrations, Cd is principally sequestered in the rhizodermis and, in the slightest way, in the cells of the endodermis. When Cd concentration in the nutrient solution was 5.0 μM, the excess of Cd was retained in the roots’ cortical regions.

### Microstructural Changes in Root Cells and Cd Deposition in Cell Walls

**Figure 6:**
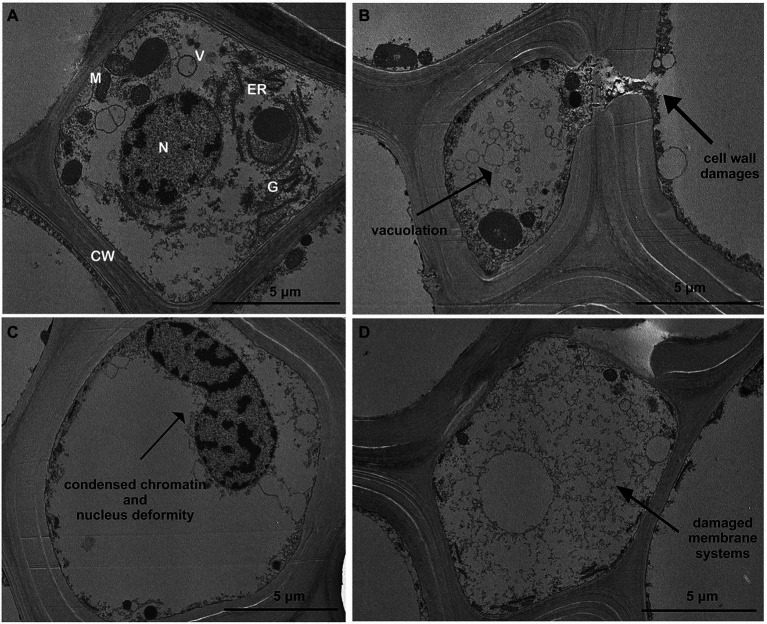
Transmission electron micrographs showing toxic effects of Cd on ultrastructure of roots (15 days from sprouting and from the start of Cd treatments). Panel **(A)** Control roots; panels **(B–D)** toxic effects observed in most of root cortical cells treated with Cd. CW, cell wall; G, Golgi apparatus; N, nucleus; ER, endoplasmic reticulum; V, vacuole; and M, mitochondria.

The root cells of control plants have a typical and organized ultrastructure with intact membranes: thin cell walls and regular shape cell organelles: mitochondria, vacuoles, Golgi apparatus, endoplasmic reticulum, cell nucleus and homogeneous cytosol (Figure 6A). In contrast to the control, root cells exposed to Cd treatments exhibited changes in the subcellular organization; the first effect visible in most of cells of the treated roots was the increased vacuolation (Figure 6B). Other alterations frequently detected in treated roots were condensed nuclear chromatin and nuclei deformities of nuclei (Figure 6C); Finally, cell wall and membrane damages (with disintegrated organelles) were also repeatedly observed in roots exposed to Cd treatments (Figures 6B,D).

**Figure 7:**
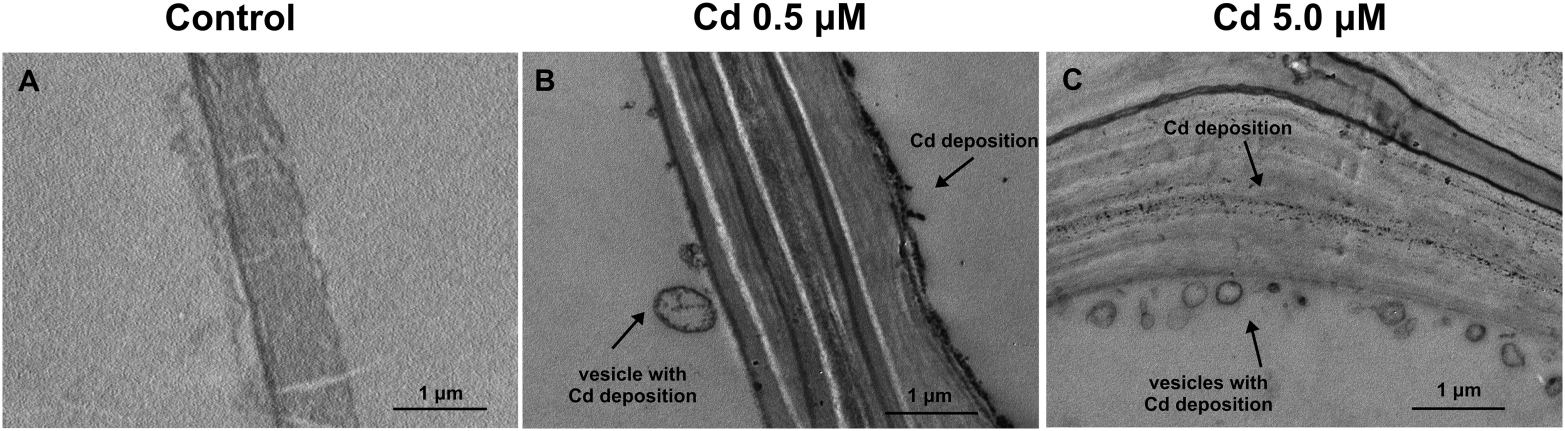
Transmission electron micrographs of root cortical cells (15 days from sprouting and from the start of Cd treatments). Panel **(A)** Control roots; panel **(B)** roots treated with Cd 0.5 μM; panel **(C)** roots treated with Cd 5.0 μM.

Moreover, cell observation of the treated plants showed an accumulation of Cd as electron-dense granules not found in roots grown in control conditions (Figures 7A–C). In fact, at the two HM concentrations, Cd appeared to be enclosed both in vesicles just behind the cell wall and within the cell wall (Figures 7B–C) despite vesicles were not constantly present. At the subcellular level, too, in the roots of the treated plants, cell walls resulted to be thicker than in the control samples (Figures 7A–C), as previously shown.

### Nicotianamine and Cd Colocalization

**Figure 8:**
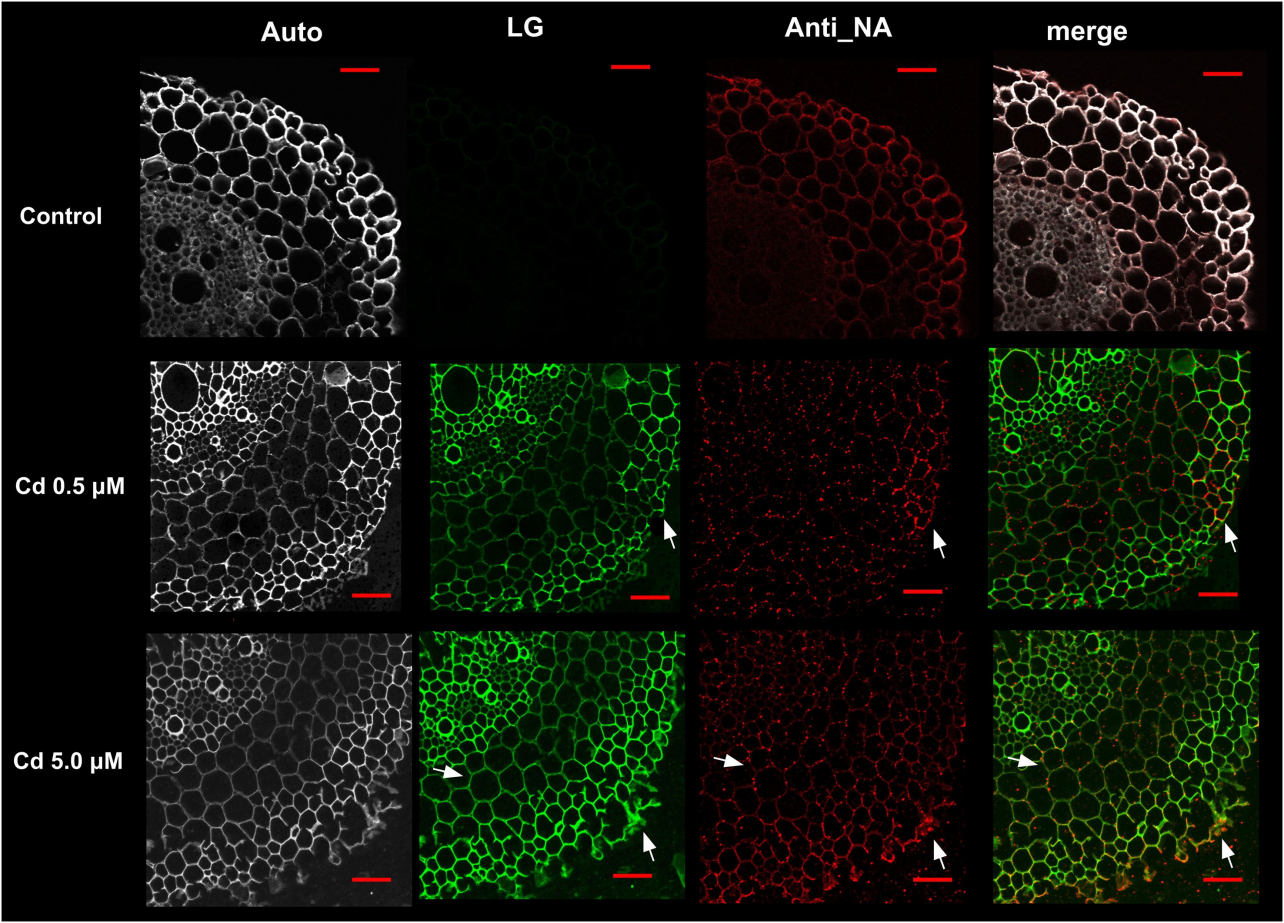
Immuno-localization of nicotianamine (NA) in roots of wheat plants grown for 15 days (starting from sprouting) in control conditions and with Cd 0.5 μM and 5.0 μM; Auto: root tissues autofluorescence; LG: root cross-sections stained with Leadmium Green AM; Anti_NA: root cross-sections labeled with antibodies directed against NA/fluorescent secondary antibodies; merge: images of green (LG) and red (Anti_NA) channels merged; white arrows indicated points of the red and green pixels colocalization. Scale bars = 50 μm.

In control samples the Nicotianamine (NA) specific labeling was uniformly distributed in the different anatomical portions of the wheat root sections (Figure 8, Anti_NA column, red color); instead, in cross-sections of root treated with Cd (both at 0.5 and 5.0 μM), the fluorescence signal appeared organized in spots as indicated by arrows (Figure 8).

Concerning the Cd distribution in root cells, after staining with Leadmium Green AM dye (LG), the fluorescence signal was localized in rhizodermis and endodermis (Figure 8, row Cd 0.5 μM) or in the cell wall of the parenchyma cells of the cortical region (Figure 8, row Cd 5.0 μM).

**Figure 9:**
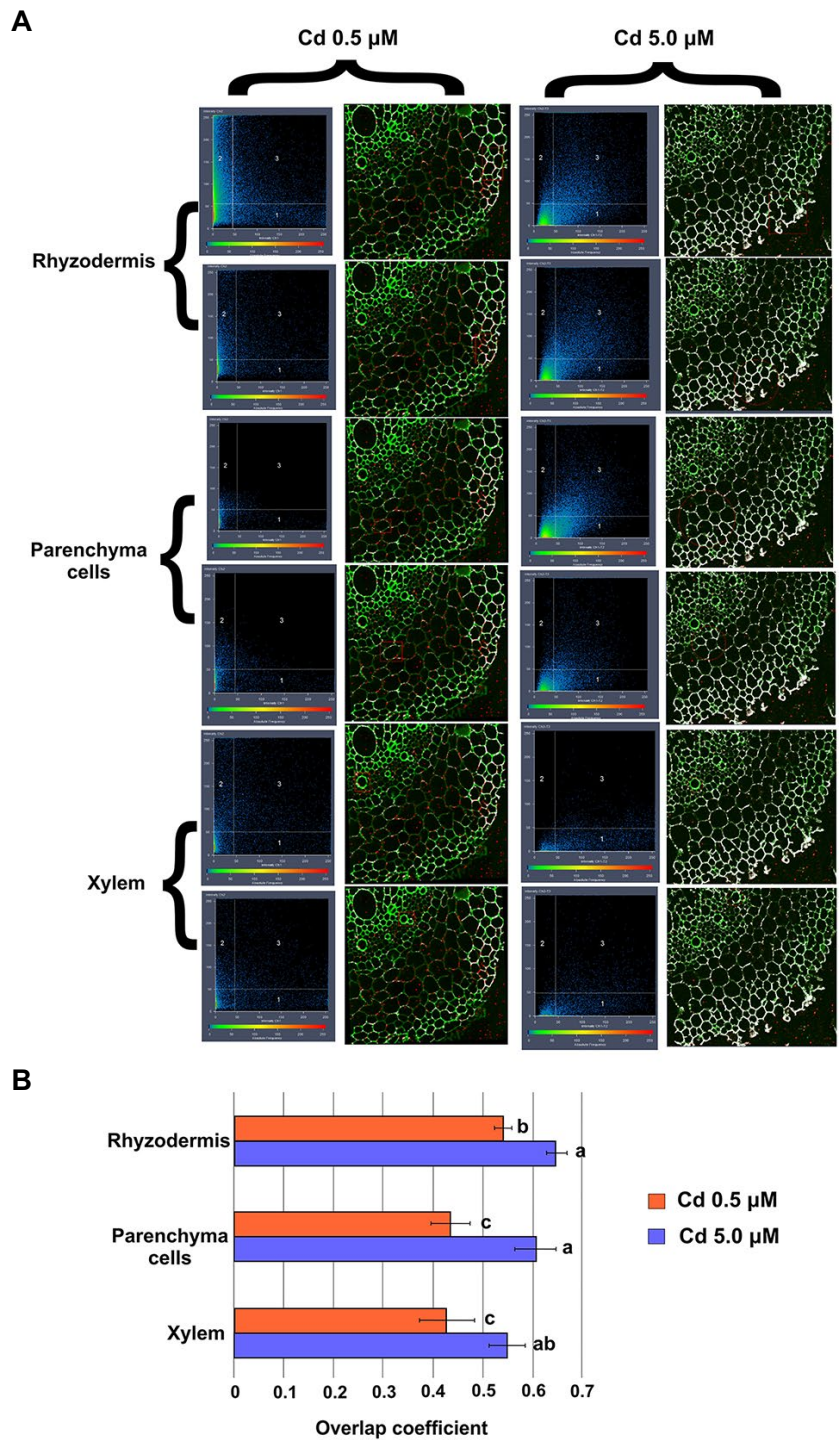
Colocalization of Cd (labeled with LG dye) and nicotianamine (labeled with anti_NA and fluorescent secondary antibodies) in root cross-sections (15 days from sprouting and from the start of Cd treatments). Panel **(A)** Representative “crosshair” scatterplots of green (LG) and red (Anti_NA) pixels intensity and colocalization in rhizodermis, parenchyma cells and xylem. Panel **(B)** Evaluation of green (LG) and red (Anti_NA) pixels colocalization by the Mander’s overlap coefficient. Data were analyzed by ANOVA followed by Tukey’s *post hoc* test. Different letters correspond to statistically different means.

Representative “crosshair” scatterplots (Figure 9A) give information related to the intensity and colocalization of green (LG staining) and red (Anti_NA) pixels in rhizodermis, parenchyma cells and xylem of the cross-sections. In roots of plants grown in presence of Cd 0.5 μM, the LG–Anti_NA colocalization was “moderate” (overlap coefficient: 0.5–0.6) in rhizodermis and “weak” (overlap coefficient: 0.4–0.5) in parenchyma cells and xylem (Figure 9B). The intensity and the LG–Anti_NA colocalization was more evident in the roots of plants treated with Cd 5.0 μM; in particular, in the cell wall of the rhizodermis and of the parenchyma cells, a “strong” (overlap coefficient: 0.6–0.7) colocalization was found, while a “moderate” (overlap coefficient: 0.5–0.6) colocalization was revealed in the xylem cell walls (Figure 9B).

### Cd Effect on Gene Expression

Quantitative real-time PCR was used for rapid and reliable quantification of mRNA transcription. However, it is crucial to choose an appropriate reference gene for an exact comparison of mRNA transcription in different samples. Among the tested genes, PP2A (contig26674) was the gene with the most constant expression in the different samples, with a coefficient variation (CV; Czechowski et al., 2005) of 0.014 (Supplementary Figure S1). Furthermore, we considered genes to be significantly up-regulated or down-regulated only in the case of fold changes greater than or equal to 2 or less than or equal to −2, respectively (Chen et al., 2007).

**Figure 10:**
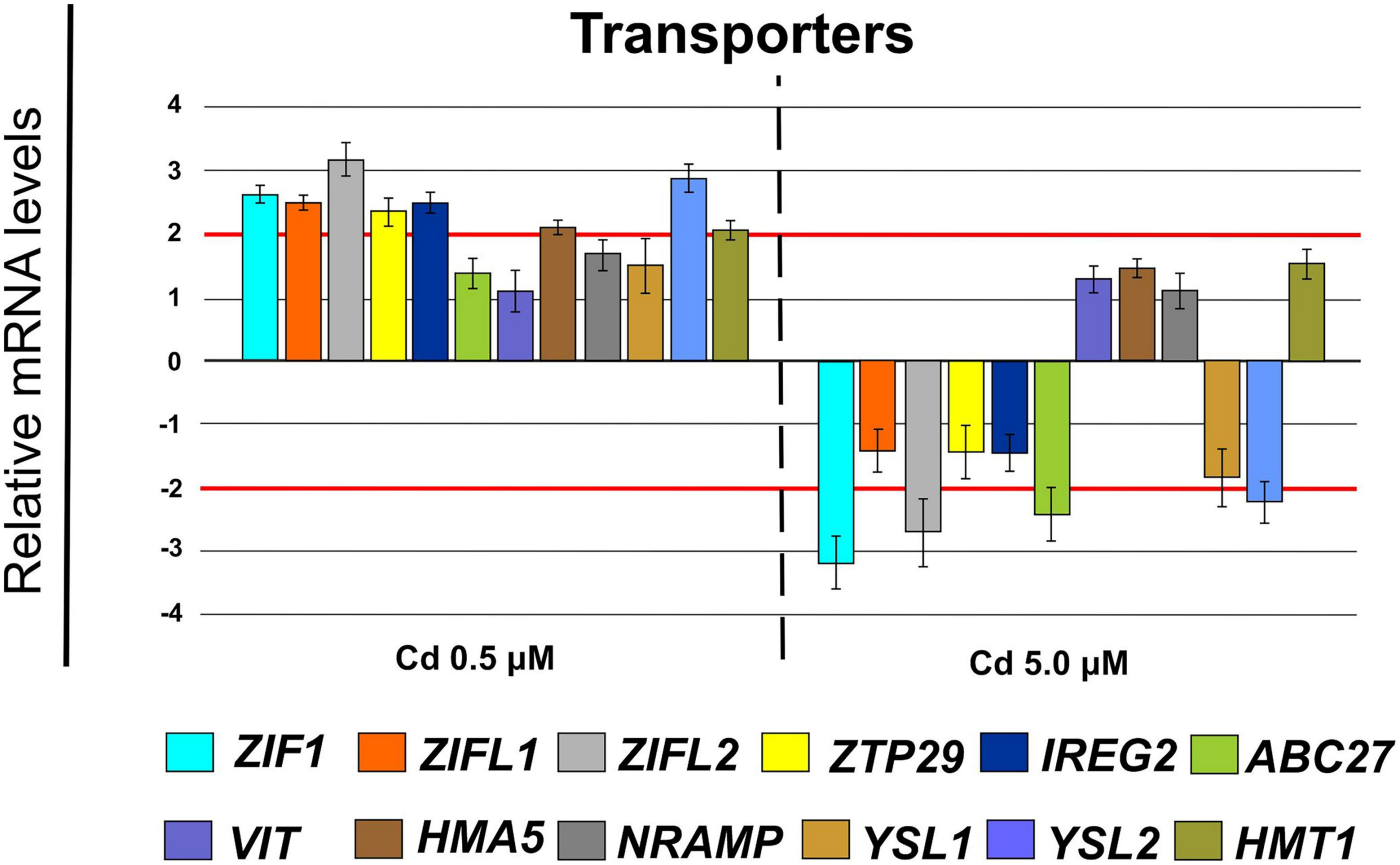
Expression analysis of genes coding for transporters in roots (15 days from sprouting and from the start of Cd treatments). *ZIF1* (*zinc-induced facilitator 1*), *ZIFL1* (*zinc-induced facilitator-like 1*), *ZIFL2* (*zinc-induced facilitator-like 2*), *ZTP29* (*zinc transporter 29*), *IREG2* (*iron regulated 2*), *ABC27* (*ATP-binding cassette 27*), *VIT* (*vacuolar iron transporter*), *HMA5* (*heavy metal atpase 5*), *NRAMP* (*Natural resistance-associated macrophage protein metal ion transporter 2*), *YSL1* (*yellow stripe like 1*), *YSL2* (*yellow stripe like 2*), *HMT1* (*heavy metal tolerance 1*). Red lines highlight the fold changes 2 and − 2 to graphically identify the genes differentially expressed.

At transcriptional level, compared with control, Cd treatment (0.5 μM) significantly up-regulated the expression of genes coding for HM transporters (Figure 10) with a fold change (FC) indicated in brackets: ZIF1 (2.65 ± 0.15), ZIFL1 (2.49 ± 0.11), ZIFL2 (3.17 ± 0.28), ZTP29 (2.39 ± 0.23), IREG2 (2.50 ± 0.16), HMA5 (2.13 ± 0.13), YSL2 (2.86 ± 0.24) and HMT1 (2.08 ± 0.16). In the presence of Cd 5.0 μM the mentioned genes were mainly not differentially expressed with only ZIF1, ZIFL2, ABC27 and YSL2 with a FC below −2.

**Figure 11:**
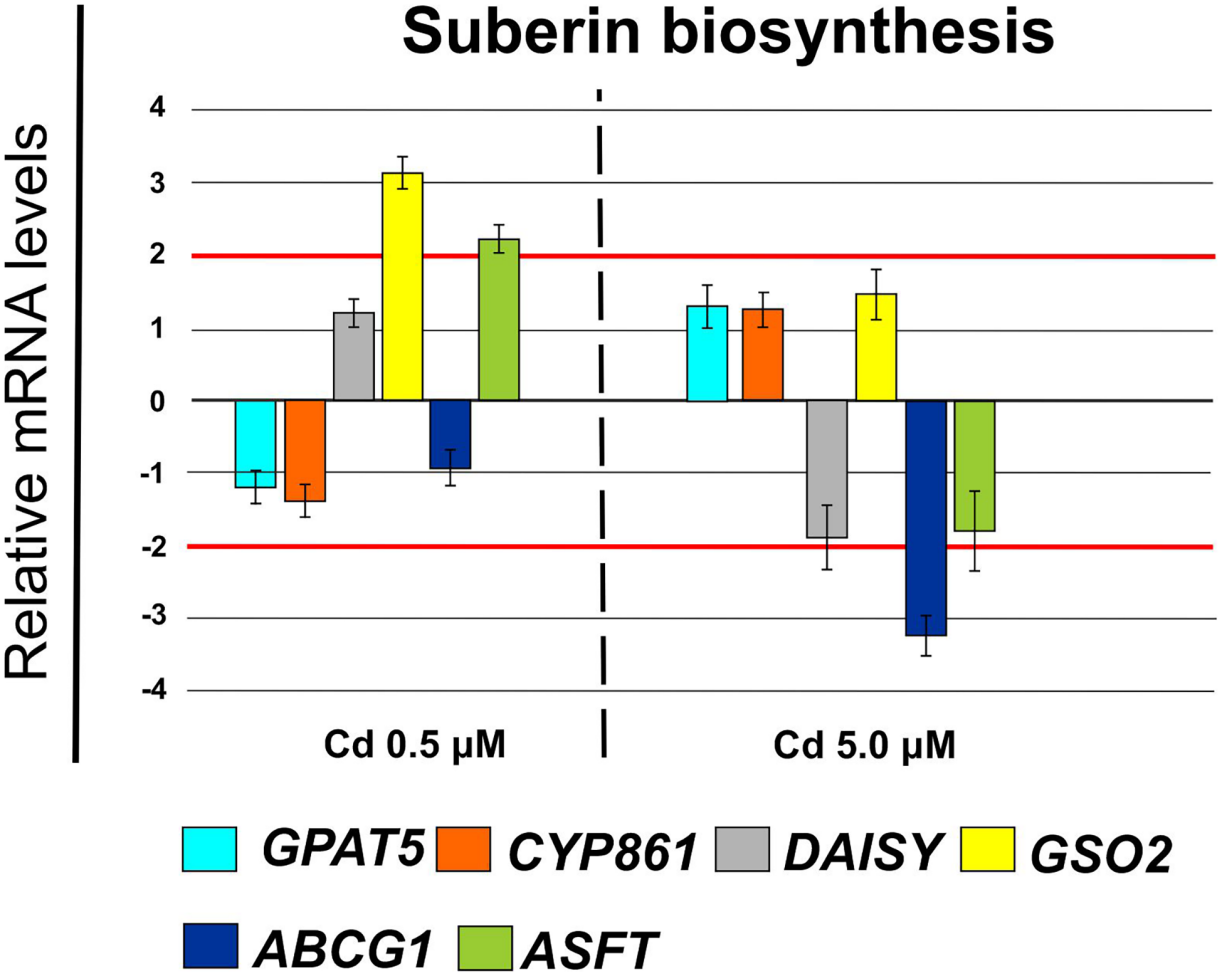
Expression analysis of genes coding for enzymes involved in suberin biosynthesis in Iride roots (15 days from sprouting and from the start of Cd treatments). *GPAT5* (*glycerol-3-phosphate acyl transferase 5*), *CYP861* (*cytochrome P450*), *DAISY* (*docosanoic acid synthase*), *GSO2* (*LRR receptor-like serine/threonine-protein kinase*), *ABCG1* (*ATP Binding Cassette Subfamily G Member 1*), *ASFT* (*Aliphatic Suberin Feruloyl Transferase*). Red lines highlight the fold changes 2 and − 2 to graphically identify the genes differentially expressed.

Furthermore, Cd at 0.5 μM concentration induced changes in cellular expression of some genes coding for enzymes involved in suberin biosynthesis: GSO2 (FC = 3.42 ± 0.21) and ASFT (FC = 2.35 ± 0.23; Figure 11). Whereas, at Cd 5.0 μM ABCG1 was clearly down-regulated (FC = −3.62 ± 0.24).

**Figure 12:**
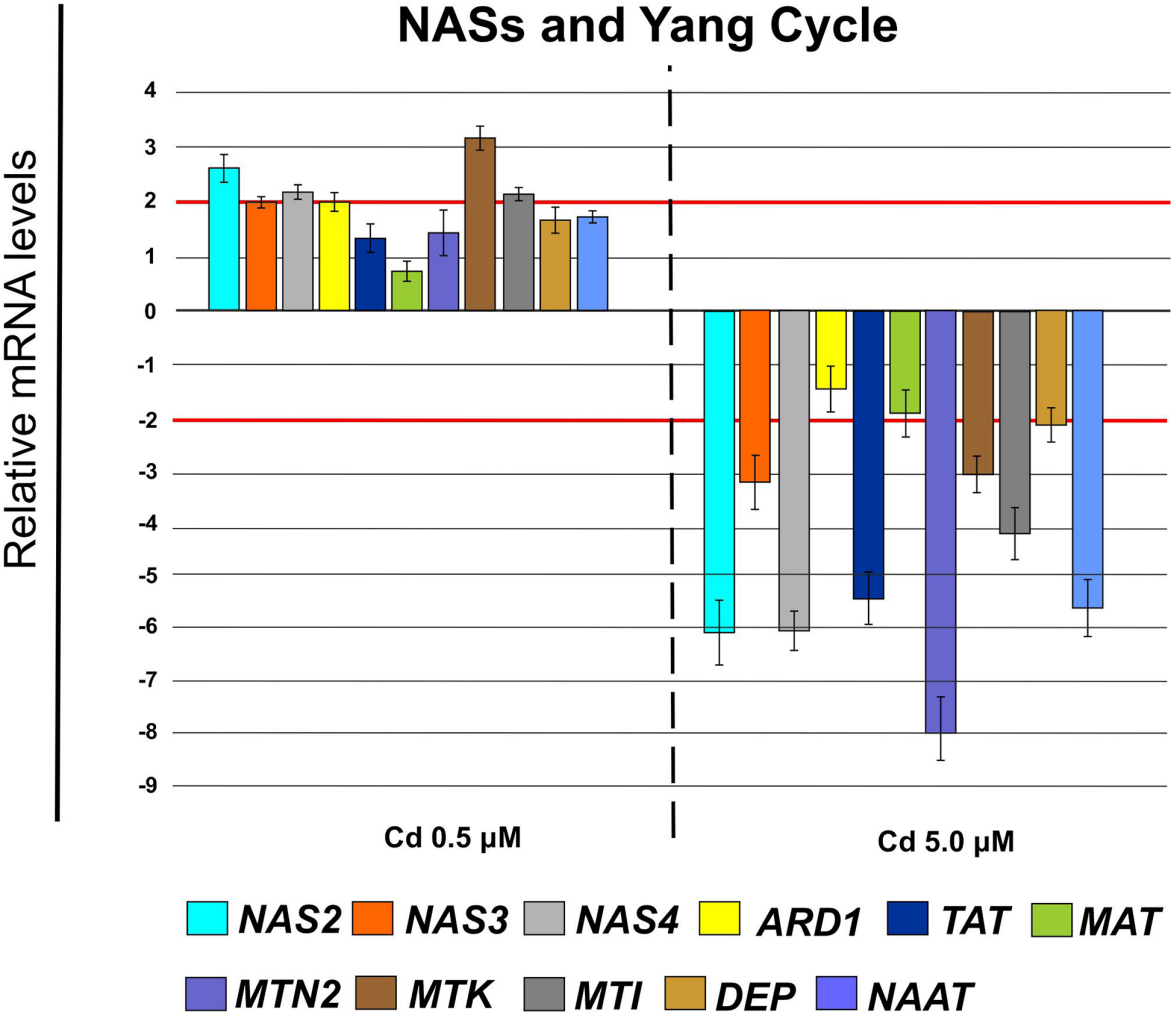
Expression analysis of genes coding for nicotianamine synthases (NASs) and enzymes involved in the pathway of Yang Cycle in roots (15 days from sprouting and from the start of Cd treatments). *NAS2* (*nicotianamine synthase 2*), *NAS3* (*nicotianamine synthase 3*) and *NAS4* (*nicotianamine synthase 4*), *ARD1* (*acireductone dioxygenase 1*), *TAT* (*tyrosine transaminase 1*), *MAT* (*S-adenosyl-L-methionine synthase*), *MTN2* (*methylthioadenosine nucleosidase 2*), *MTK* (*S-methyl-5-thioribose kinase*), *MTI* (*5-methylthioribose kinase 1*), *DEP* (*dehydratase/enolase/phosphatase*), *NAAT* (*nicotianamine aminotransferase*). Red lines highlight the fold changes 2 and − 2 to graphically identify the genes differentially expressed.

Cd 0.5 μM determined an up-regulation of the genes coding for nicotianamine synthases NAS2 (FC = 2.76 ± 0.19), NAS3 (FC = 2.10 ± 0.10) and NAS4 (FC = 2.25 ± 0.12; Figure 12) and of a few genes coding for enzymes involved in Yang Cycle as: ARD1 (FC = 2.06 ± 0.21), MTK (FC = 3.31 ± 0.20) and MTI (FC = 2.25 ± 0.19; Figure 12) while Cd 5.0 μM down-regulated substantially all these genes except ARD1 (Figure 12) which remains below the threshold of −2.

**Figure 13:**
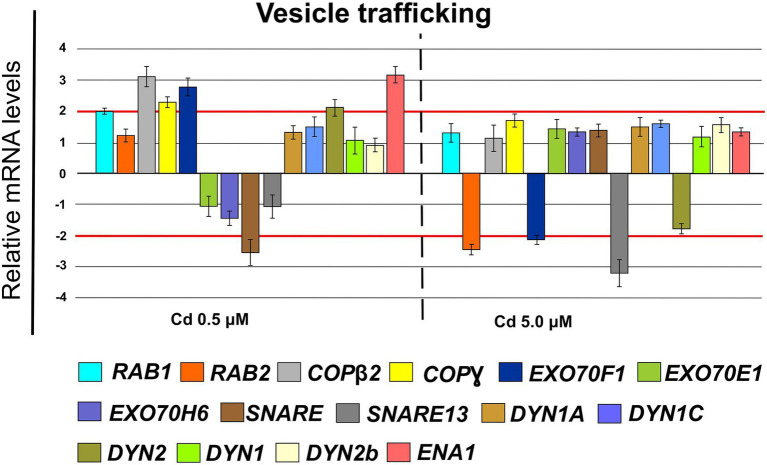
Expression analysis of genes coding for proteins involved in the vesicular trafficking in roots (15 days from sprouting and from the start of Cd treatments). *RAB1* (*Ras-related in brain 1*), *RAB2* (*Ras-related in brain 2*), *COPß1* (*coat protein ß1*), *COPϒ* (*coat protein ϒ*), *EXO70F1* (*exocyst component of 70 kDa F1*), *EXO70E1* (*exocyst component of 70 kDa E1*), *EXO70H6* (*exocyst component of 70 kDa H6*), *SNARE* (*soluble N-ethylmaleimide-sensitive factor attachment receptor*), *SNARE13* (*soluble N-ethylmaleimide– sensitive factor attachment receptor 13*), *DYN1A* (*dynamin 1A*), *DYN1C* (*dynamin 1C*), *DYN2* (*dynamin 2*), *DYN1* (*dynamin 1*), *DYN2b* (*dynamin 2b*), *ENA1* (*nicotianamine exporter 1*). Red lines highlight the fold changes 2 and − 2 to graphically identify the genes differentially expressed.

Finally, in roots treated with Cd 0.5 μM, among the genes coding for proteins involved in vesicle trafficking, we found the up-regulation of the following genes (Figure 13): RAB1 (FC = 2.00 ± 0.12), COPß2 (FC = 3.35 ± 0.29), COPϒ (FC = 2.45 ± 0.21), EXO70F1 (FC = 3.00 ± 0.28), DYN2 (FC = 2.30 ± 0.26), ENA1 (FC = 3.21 ± 0.27); while, the gene coding for the SNARE protein resulted down-regulated (FC = −2.65 ± 0.35; Figure 13). When roots were treated with Cd 5.0 μM, the threshold of −2 is only exceeded in down-regulation by RAB2, EXO70F1 and SNARE13; all the analyzed genes resulted not differentially expressed (Figure 13).

### Cd Stimulated Suberin Deposition

**Figure 14:**
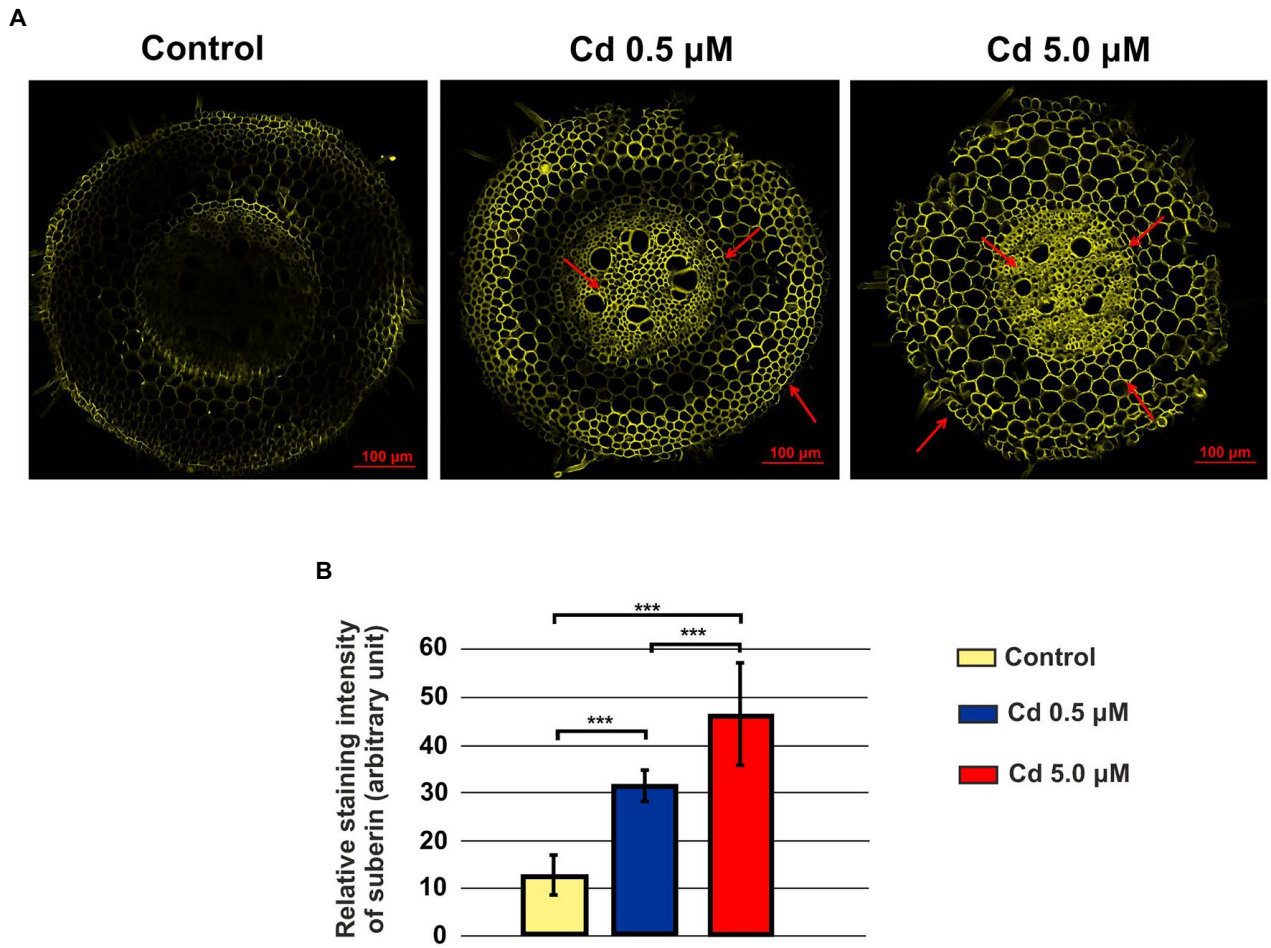
Suberin in roots visualized by fluorescent imaging (staining with Fluorol Yellow 088). Panel **(A)** Yellow fluorescence in the images represents the binding of the dye to suberin; control: a faint but not bright signal was observed; Cd 0.5 μM: fluorescence is localized in rhizodermis, endodermis and vascular cylinder (red arrows); Cd 5.0 μM: fluorescence is also localized in the cell wall of the parenchyma cells of the cortical region (red arrows). Panel **(B)** Quantification of the relative intensity of the fluorescence signals. *T*-test value of *p* < 0.001***.

Suberin was clearly detected in rhizodermis, endodermis and vascular cylinder of roots treated with Cd 0.5 and 5.0 μM (Figure 14A); suberin deposition was also detected in the cell wall of the parenchyma cells of the cortical region at the higher Cd concentration (Figure 14A). The quantification of the relative intensity of the fluorescence signal showed a statistically significant increase of suberin with Cd treatments suggesting that Cd stress induced suberization (Figure 14B).

## Discussion

Durum wheat Iride plants were hydroponically grown in the absence or presence of cadmium at two concentrations. The Cd treatment significantly affected the root anatomy, both at 0.5 and 5.0 μM. At the macroscopic scale, an increase of the average number of tips was recorded (Figures 1A–C), while, at the higher Cd concentration of 5.0 μM, a rise of the primary root length and a darkening of the root were detected (Figures 1A,D). An increase in the number of tips in response to Cd treatment was already reported for *Arabidopis thaliana* (Bahmani et al., 2016), radish (Vitòria et al., 2003), barley (Ďurčekovà et al., 2007), sorghum (Kuriakose and Prasad, 2008) and maize (Seregin and Ivanov, 2001), suggesting two possible explanations. The first one, proposed by Bahmani et al. (2016), provides that the Cd accelerates the maturation of the cells involved in root hair fate; the second, suggested by Lux et al. (2011), assumes that the induction of lateral roots is an adaptive avoidance to Cd. In fact, roots exposed to agar layers containing Cd promote the production of lateral roots on the opposite side to the Cd. Symptoms of Cd toxicity were visible in roots at Cd 5.0 μM as a root browning (Figure 1A). A similar effect has been reported in several works for different plant species treated with Cd (Mahmood et al., 2007; Malecka et al., 2012; Moreira et al., 2020). The frequently observed root browning, as the main symptom of HM toxicity (Ni, Cu, Cd and Pb), is usually correlated with Cd-induced oxidative stress (Ai-jun et al., 2007) and with enhanced suberization, which may limit Cd uptake (Nada et al., 2007; Parrotta et al., 2015). Moreover, root lignification induced by some HMs like Cd, also improves resistance to water flow within root. In fact, the increase of the primary root length (that we observed in roots treated with Cd 5.0 μM) can be associated both with water deficit (root grow to optimize its access to water supply; Dinneny, 2019) and hormonal balance alteration induced by the oxidative stress at higher Cd concentration (Atici et al., 2003; Pèrez-Chaca et al., 2014). In comparison to control, root diameter in plants treated with Cd did not change significantly (Figure 1E), but ultrastructural analysis of cross-sections showed changes in the root system architecture; in fact, Cd treatments induced a significant pericycle and xylem cell wall thickening (Figures 2A–G). This finding is in accordance with the work of Pèrez-Chaca et al. (2014) who reported hypertrophy of the vascular tissues with thickened walls in Cd-treated roots of soybean (*Glycine max* L.) and linked these anatomical alterations with metal retention in the cell wall. In addition, Guo et al. (2021) investigated root cell wall modifications induced by Cd in the NHE (non-hyperaccumulating) and in HE (hyperaccumulating) of *Sedum alfredii*, reporting that in NHE plants the thickness of the root cell walls was twofold than HE leading to more Cd trapped. Moreover, cell wall thickening of vascular elements in roots determines the reduction of the vascular lumens also observed in Cd-exposed pea roots (Rodrìguez-Serrano et al., 2006). Our data related to the Cd content in roots, shoots and grains (Figures 3A–C) confirmed that durum wheat (cv. Iride) retains more Cd in root and consequently translocates small quantities to the aerial parts of the plant. The same model to explain the restricted translocation of Cd to the aboveground organs has been proposed by Harris and Taylor (2013) in the low grain-Cd isogenic line; they supposed greater sequestration of Cd in the root symplasm, sequestered into chemical compounds. As a consequence, they reported that the Cd concentration in shoots and grains is lower than the concentrations observed in genotypes that are not able to sequester Cd in roots as the high grain-Cd isogenic line. Since treatment with Cd induces iron deficiency in several plant species influencing root morphology (for example, the increase of primary root length and root branching; Jin et al., 2008), we also investigated if Cd treatments affect the uptake of some mineral elements. Our data indicate that the Cd treatments did not alter the iron root content, while some variations were detected for manganese and zinc both in root and shoot (Figures 4A,B). In roots, manganese content increased only at 0.5 μM Cd, while in shoots, Mn content decreased in respect to the control (Figures 4A,B). Reductions in Mn uptake and accumulation in the shoots and roots have been reported in different plants, including durum wheat (Jalil et al., 1994), maize (Yang et al., 1998) and barley (Wu et al., 2003), when grown in Cd-polluted media; in these previous works authors speculated a competitive interaction between Mn and Cd since, probably, they use a common transport systems in plants. Instead, zinc content was affected by the Cd treatments both in root and in shoot: in roots, Cd treatments induced Zn increase while in shoots, only the treatment with Cd 5.0 μM induced augmentation of its content (Figures 4A,B). Indeed, Zn and Cd cross interactions are well recognized and widely reported: these two metals show a high level of chemical similarity and their divalent cations uptake is regulated by modifying the root-specific expression pattern of ZIPs transporters (Palusińska et al., 2020). This information is in agreement with our data because, among the genes coding for transporters, in roots treated with Cd 0.5 μM we found significantly up-regulated of the following genes: ZTP29 (Zinc transporter 29), ZIF1 (Zinc-induced facilitator 1), ZIFL1 (Zinc-induced facilitator-like 1) and ZIFL2 (Zinc-induced facilitator-like 2; Figure 10). On the contrary, when roots grew with Cd 5.0 μM, most of the genes were down-regulated after Cd treatment (Figure 10) which indicates cellular toxicity as proposed by Cui et al. (2020). Also, Ram et al. (2019), in their work of characterization and expression of MTP (Metal Tolerance Proteins) genes under HM stress conditions (concentrations from 50 to 100 μM), found that among the differentially expressed genes, most of them showed down-regulation in roots and up-regulation in shoots indicating that extended HM treatments can significantly alter plant physiology. We observed some effects of metal toxicity at the ultrastructural level with cell wall damages, vacuolation, highly condensed nuclear chromatin and nuclei deformities (Figure 6). For a number of HMs, including Cd and Zn, increased vacuolation is reported as a mechanism that cells induce to prevent the free circulation of HM ions in the cytosol (Hall, 2002; Liu and Kottke, 2004). Most studies on Cd toxicity described nuclear changes such as chromatin condensation and DNA fragmentation since Cd exposure, among the proposed mechanisms, causes DNA strand breakage and inhibition of DNA repair (Niekerk et al., 2021). Among these toxic effects, condensed chromatin is reported to reduce gene expression since when chromatin is more condensed, it is harder for transcription factors and DNA binding protein to access DNA (Niekerk et al., 2021).

So, according to the reported data, the wheat cultivar Iride trapped Cd in root avoiding translocation in the aboveground portions of the plant. As disclosed in other works, plants that accumulate Cd in roots usually compartmentalize the HM in endoderm and cortical regions of roots; these regions act as a filter to avoid Cd translocation in xylem vessels and then to the shoot (Parrotta et al., 2015). We observed a similar strategy in durum wheat roots; in roots treated with Cd 0.5 μM, Cd was localized in rhizodermis and endodermis (Figures 5C,D) while, after the 5.0 μM Cd treatment, the HM was also localized in parenchyma cells of the cortical region (Figures 5E,F). These data indicate that the ability of roots to retain Cd in such filter tissues is an efficient strategy to sequester the HM and to limit its diffusion in upper organs.

To avoid Cd toxicity, plants have developed several strategies for Cd exclusion, such as chelation, binding Cd to the cell wall, sequestration into vacuoles, or limiting Cd accumulation in tissues/organelles (Clemens et al., 2013). For example, in barley, Cd-resistant genotypes accumulate more Cd in the cell wall (Wu et al., 2003); on the contrary, in rice, the strategy to accumulate Cd into vacuoles was observed (Miyadate et al., 2011; Wei et al., 2021). Our ultrastructure investigation showed Cd deposition in multi vesicles located near or inside the cell walls (Figures 7B,C) and that the root cell walls of Cd-treated plants appeared thicker than control samples (Figures 7A–C). Predominant accumulation of Cd in the root cell walls was also reported in *Spartina alterniflora* (Pan et al., 2012) and *Kandelia obovata* (Weng et al., 2012) treated with different Cd concentrations. In bread wheat (*T. aestivum* L.) Cd was trapped in the cell wall of roots by endodermal suberization (Wu et al., 2019). In our case, we observed cell wall thickened, and the up-regulation of some key genes involved in suberin biosynthesis (GSO2 and ASFT; Figure 11). Moreover, a statistically significant increase of suberin was directly observed and quantified in root tissues treated with Cd both 0.5 and 5.0 μM (Figures 14A,B). The accumulation of suberin was similarly detected in roots of the medicinal plant *Merwilla plumbea* exposed to Cd; this process has been explained as a protective response against the HM penetration in the cells (Lux et al., 2011). In fact, among the mechanisms for tolerating HMs, extracellular compartmentalization far away from vital cellular components is another efficient strategy to limit HM toxicity. The cell wall can effectively act as a compartment for Cd resistance, but it is clear that it requires the cooperation of different metabolic activities: e.g., the synthesis of molecules capable of binding Cd sequestering it, the transport of the formed complexes and the immobilization of Cd into the cell wall (Parrotta et al., 2015). Concerning the production of specific chelating agents, in previous work (Aprile et al., 2018), we reported the synthesis of the phytochelatin nicotianamine (NA) and the activation of genes involved in the Yang Cycle in durum wheat roots treated with Cd. Also, in the cultivar Iride treated with Cd 0.5 μM, the up-regulation of the genes coding for the nicotianamine synthases 2, 3 and 4 (NAS2, NAS3 and NAS4, Figure 12) and of the genes involved in the Yang Cycle (ARD1, MTK and MTI, Figure 12), together with the observations of immuno-localization of the NA (Figures 8, 9) indicate production of NA that in turn chelates Cd in roots. Extensive physiological and molecular studies have demonstrated the role of NA as Cd-chelator for Cd-tolerance in plants (Kim et al., 2005; Koen et al., 2013; Meyer et al., 2015). In fact, the detection of NA in the apoplast (Figure 8) is in agreement with the observations reported in other published works (Van der Vliet et al., 2007; Curie et al., 2009; Isaure et al., 2015; Haider et al., 2021): NA apoplastic pool, by forming complex, can sequester Cd through the formation of apoplastic barriers, such as the suberin, this allows the regulation of Cd uptake and translocation in the xylem. NA is a biosynthetic precursor of mucigenic acids (MAs), also important for translocating divalent metals. This translocation activity for the subcellular distribution of divalent cations is achieved through intracellular vesicle trafficking (Nozoye et al., 2019); indeed, in the images obtained from our ultrastructure investigation, Cd deposition was revealed in multi vesicles and near or inside the cell wall (Figures 7D,F) following the Cd treatment. Anymore, these observations were supported by the expression data of genes coding for genes involved in vesicular trafficking (Figure 13); RAB1, COPß2, COPϒ, EXO70F1, DYN2, and ENA1 resulted up-regulated in roots treated with Cd 0.5 μM. As for mammals, RAB1, COPß2, COPϒ and DYN2 are the major molecular players in vesicle-mediated protein transport (Hwang and Robinson, 2009). EXO70F1 and ENA1 are involved in exocytosis in many plant tissues; EXO70 is a conserved subunit of the exocyst complex that mediates the bind of exocytic vesicles with the plasma membranes (Fendrych et al., 2013). ENA1 is mainly expressed in roots and exports NA (nicotianamine) through vesicular trafficking (Nozoye et al., 2019). On the other side, the gene coding for the SNARE protein resulted down-regulated (Figure 13); however, in the description associated with the annotation of this gene was reported the caption “SNARE associated Golgi protein family” suggesting that the gene codes for a SNARE protein involved in the fusion of vesicles with Golgi membranes; in this perspective, its down-regulation makes sense to favor vesicular transport toward the plasma membrane and exocytosis. Among the up-regulated genes, we also found the genes YSL2, IREG2, HMA5 and HMT1 (Figure 10); the last three are well-known genes coding for transporters involved in Cd detoxification and tolerance in *Arabidopsis* (Long et al., 2010; Park et al., 2012; Wu et al., 2012) and in durum wheat (Sabella et al., 2021) while YSL2 codes for a nicotianamine transmembrane transporter (Conte et al., 2013). Taken together, the presented data indicate that, in the cv. Iride roots, Cd is immobilized in the suberized cell walls through the bond with nicotianamine and the transfer *via* vesicles of the NA-Cd complex.

## Conclusion

Plants can accumulate toxic amounts of Cd, but a low Cd concentration in the wheat staple food is desirable. Wheat plants (*cv*. Iride) reduce Cd translocation to the aerial organs of the plant by the Cd sequestration in roots. Our findings indicate the mechanisms activated to achieve this compartmentalization: thickening and suberization of root cell walls, synthesis of the phytosiderophore nicotianamine (NA) which chelate Cd, exocytic vesicular transport of the Cd-NA chelates to the cell walls. These new findings could help address future breeding programs to decrease Cd concentration in wheat through genetic improvement.

## Data Availability Statement

The original contributions presented in the study are included in the article/Supplementary Material, and further inquiries can be directed to the corresponding author.

## Author Contributions

AA, ES, and LB planned the experimental design and wrote the manuscript. AA, ES, and MV grew plants in hydroponic conditions and evaluated root morphometric data. AA carried out the chemical analysis on cadmium and micronutrients concentration in tissues. ES achieved scanning electron microscopy and laser-scanning microscopy experiments. EP, EC, and BT conducted the electron transmission microscopy experiments. AL processed samples for mRNA extraction and qRT-PCR analysis. All authors contributed to the article and approved the submitted version.

## Conflict of Interest

The authors declare that the research was conducted in the absence of any commercial or financial relationships that could be construed as a potential conflict of interest.

## Publisher’s Note

All claims expressed in this article are solely those of the authors and do not necessarily represent those of their affiliated organizations, or those of the publisher, the editors and the reviewers. Any product that may be evaluated in this article, or claim that may be made by its manufacturer, is not guaranteed or endorsed by the publisher.
