# Supplementary figures and images for: Effects of Cadmium on Root Morpho-Physiology of Durum Wheat
Source: Front Plant Sci. 2022 Jun 23;13:936020. doi: 10.3389/fpls.2022.936020 (PMC9260267; doi:10.3389/fpls.2022.936020)

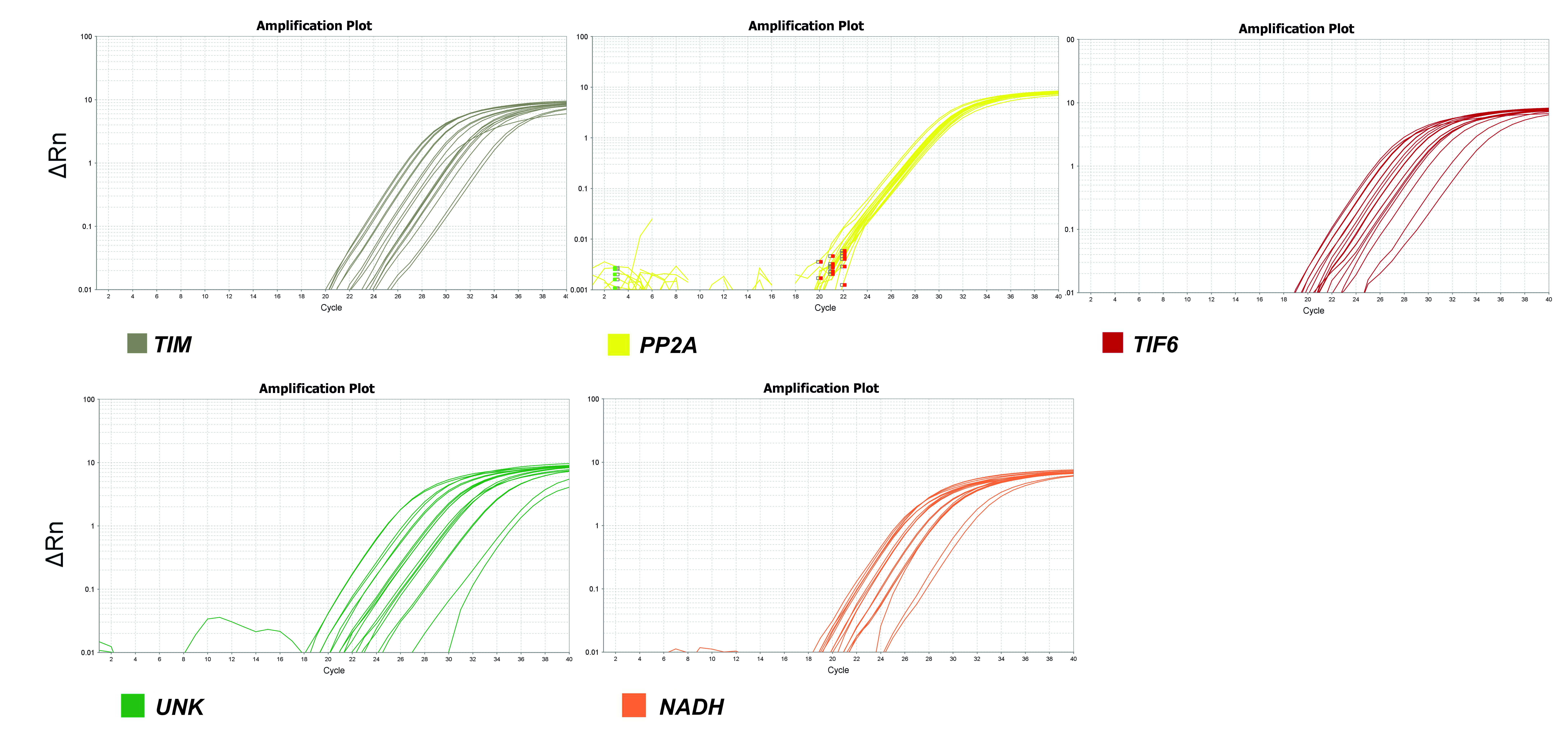

Supplement: Supplementary file 1 [file Image_1.JPEG]
